# Not doomed: sociology and psychiatry, and ignorance and expertise

**DOI:** 10.1192/bjb.2022.60

**Published:** 2023-04

**Authors:** George Ikkos

**Affiliations:** Royal National Orthopaedic Hospital, London, UK

**Keywords:** Aetiology, history of psychiatry, sociology of knowledge, psychiatric epistemology, clinical services

## Abstract

This paper presents and responds to *On the Heels of Ignorance*, a sociological study which identifies five fundamental epistemological paradigm changes in American psychiatry in the service of its survival and details several tactics that have been employed to facilitate these professional reinventions. Issues raised in this presentation include the relationship between psychiatry, society and the state, and the nature and significance of psychiatric expertise. The dynamic of these relationships and the complexities of the required expertise create their own challenges for the advancement and professional accountability of the specialty. The conclusion suggests some future imperatives.

According to Whooley, an Associate Professor of Sociology at the University of New Mexico, there is a growing body of sociological research on ‘ignorance’, including the ignorance of the professions.^1^ He suggests that from the perspective of this field, psychiatry serves as fertile ground for study. He neatly summarises his argument as follows:

‘Psychiatry has been shaped less by the knowledge it has secured and more by the ignorance it cannot resolve. Indeed, the most striking feature of psychiatry's history is how little progress it has made in resolving its basic questions. Psychiatry has changed, there can be no doubt. But how far has it really come?’ (p. 197).^1^

Some will accuse Whooley of being yet another in a long line of anti-psychiatrists, but he concedes the concept of mental illness when he states ‘We [society] have abdicated our duty to those with serious mental illness’ (p. 201) and affirms the legitimacy of biomedical research on mental illness when he writes that ‘The parallel with previous reinventions does not mean the neuroscientific vision of psychiatry will necessarily fail, that it will succumb to the same fate as its predecessors. It is far too early to tell’. Militant defenders of psychiatry may even derive some pleasure from reading that ‘Foucault is known for playing fast and loose with the historical record. He paints vivid pictures and provocative arguments but often at the expense of rigour’ (p. 220).

## Outline of issues

Whooley's thesis is that during the 200 years he surveys, the rest of American medicine has reinvented itself once whereas psychiatry had done this five times. Furthermore, whereas the bulk of medicine reinvented itself early in the 20th century in response to clinically relevant advances in laboratory science, whenever psychiatry changed its basic scientific paradigm it was in the absence of similar progress. One of the most interesting aspects of the book is that, at each time of change, the emerging new psychiatric leadership has openly acknowledged the failure of the previous paradigm. Faced with existential threat it reinvented the profession based on promises rather than evidence, wishful thinking or snake oil if you wish.

The imperatives of professionalism require that every medical specialty accounts for its choices and actions to the public.^2^ It is a thesis of Whooley that some leaders and members of the profession have acted hubristically and this has produced problematic results, including some of psychiatry's worse abuses. Of course, in stating this, it is not necessary to assume that the bulk of American psychiatrists set out to lie or hoodwink the public. Just that they selected among alternatives each time a strategy that seemed ‘realistic’ in the circumstances. One that would allow them to secure research funding, offer some service to their patients, make a living, feed their families, etc. If so, they would not be acting any differently from their colleagues in other countries, or in other medical specialties in the USA. The value of sociological analysis is to illuminate not individual motivation but the forces that bear on it and communal action, and their consequences.

In this paper, Whooley's argument will be presented first. The role of psychiatry in society and the relevance of national politics will be discussed next. Important differences between British and US psychiatry will be acknowledged and their implications for the future will be explored. The third section will reflect on the nature of psychiatric expertise, followed by further discussion and conclusions. Although British psychiatry may not have been subject to the same extremes and dramatic shifts that Whooley documents, it has had its own changes of direction,^3^ sometimes ideological,^4,5^ and we should take the opportunity to reflect and learn from the American experience.

## The psycho-politics of ignorance

Whooley's research strategy has been to survey the writings of the psychiatric ‘literary elite’ (p. 23)^1^ through the contents of the *American Journal of Psychiatry* and its predecessors. As a result, he identifies these five phases:

1. asylum psychiatry: ‘The general superintendence of all their departments’ (Ch. 1)
2. psychobiology: ‘Unruly ignorance and pragmatic eclecticism’ (Ch. 2)
3. psychoanalysis: ‘Ignorance repressed’ (Ch. 3)
4. community psychiatry: ‘It takes a community to raise a profession’ (Ch. 4) and
5. diagnostic psychiatry: ‘Profession of the book’ (DSM-III and after) (Ch. 5).

Asylums were hailed as therapeutic institutions but, despite some patient turnover, ended up being ‘warehouses’ for the variously excluded. Furthermore, superintendents fiddled with statistics to conceal their failure to live up to promises of cure. Once this was discovered, municipal and regional legislators lost confidence and withheld funding, especially during economic downturns. Psychobiology, led by the Swiss immigrant psychiatrist Adolf Meyer, embraced a broad eclectic approach that allowed psychiatric practice to escape the confines of the failing asylums, but reached an impasse in terms of research and service development. A proposed key tool was the ‘life chart’, but its use varied and the model lacked theoretical and methodological rigour. Psychoanalysis offered theoretical conviction and clinical focus through investigation and interpretation of the unconscious in the privacy of the consulting room, but any scientific foundation was weak, philosophically even weak in its own terms according to Adolf Grünbaum.^6^ An unsavoury side of this phase has been that contrary to Freud's views and contemporary European practice, psychiatrists maintained pecuniary privilege by excluding all non-medics from the American Psychoanalytic Association for decades.

If psychoanalysis narrowed the focus of psychiatry to the sometimes interminable, sometimes arbitrary exploration of transference and countertransference for those who could afford to pay, community psychiatry attempted to better serve social justice and practicality with an ill-defined lionisation of community-based services. When community mental health centres were widely perceived to have failed and President Ronald Reagan, between 1981 and 1989, implemented massive cuts to public services, this phase came to an end. Faced with this reality American psychiatry found itself defenceless. In such circumstances, DSM-III was an urgent attempt to proclaim medical legitimacy and secure funding for research and clinical care. It has achieved some success,^7^ but the abandonment of DSM-5 by the National Institute of Mental Health (NIMH) in favour of the Research Domain Criteria (RDoC) just days before its publication in 2013 broadcast its failure to achieve its main strategic objective. Whooley argues that DSM was incredibly important in shoring up the profession's authority at the time, but that the strategy of asserting professional authority vis-à-vis classification may have run its course (as evident in the challenges the DSM-5 revision faced). I would suggest that any ‘political’ success DSM may have achieved has been at the expense of epistemological rigour and clinical sensibility. These were apparent even at the launch of DSM-III and have extracted a great cost to the reputation of the profession.

Space does not allow me to go into further detail on Whooley's theoretical analysis, but in his concluding chapter (Ch. 6) he offers a typology of tactics employed in the service of survival: appeal to exemplars, appropriation, bandwagoning, deflecting blame onto the object, mystification, naming the object, rescaling the object, retrenchment, forging new alliances and shifting arenas. These are well worth looking up if psychiatrists are to subject ourselves to rigorous scrutiny.

By the end of this story, according to Whooley, some psychiatrists had caught on to the bankruptcy of the strategy of repeated reinvention and, led by Robert Spitzer and Allen Frances, successfully revolted against yet another change of paradigm during the DSM-5 revision process.

## Psychiatry, society and the state

Leaving aside the question of whether the rest of medicine is as pristine as the above summary might intimate, some of the charges against American psychiatry could be disputed. For example, the charge against community psychiatry that it lacked evidence may be judged as harsh. As Whooley details, the small but influential Group for the Advancement of Psychiatry (GAP), which was pivotal to this phase, was aware of the limited evidence available at the time and played a major role in the establishment of NIMH, which in its early years focused primarily on service-related research. Unfortunately, as the author makes clear, such aspirations fell prey to Ronald Reagan's neoliberal revolution and its fundamentalist faith in market solutions and later to technology in the Decade of the Brain. Could psychiatrists have done more under the circumstances? Perhaps. I think so.

Though there is ground for disagreement, in my view Whooley paints a vivid picture and his core argument about repeated change of paradigm driven significantly by wishful thinking and political-professional interest will likely stand up to the scrutiny of time. For example, others have commented on the abrupt changes in direction in American psychiatry too.^8^ Whether American or not, psychiatrists will do well to familiarise themselves with it. We may all learn something despite our differences and through those differences. For example, a difference is that although Margaret Thatcher preceded and perhaps inspired Ronald Reagan in the neoliberal assault on the state, Britain has had a stronger socialist tradition and welfare state and even Conservative politicians have now abandoned her open crusade against the National Health Service (NHS) and have been talking it up since the turn of the millennium. As a result, services seem to have held up better,^9^ though not well either.^10^ The threat is not over though, and these days the NHS is being privatised by stealth rather than public announcements and confrontation. A British Medical Association report on the independent healthcare sector in the NHS in 2019 found that ‘As a proportion of the DHSC [Department of Health and Social Security] budget, independent sector healthcare provision remains high compared to historic levels’.^11^ Its unhappy experience of emulating US healthcare strategies^12^ suggests that the UK should seek to actively distance the values and practices of service design and management from those of its transatlantic friends. This has mostly not been so. Even the last Labour government looked eagerly to the US for inspiration on health service management^13^ and, for example, relied on the private finance initiative (PFI) for hospital infrastructure development, at high ultimate expense.

Whooley reserves his strongest condemnation not for psychiatry but for the scandalous neglect of the severely mentally ill by broader society in the USA:

‘The most damning evidence of our indifference, however, comes from how we invest our resources, or more accurately, how we decide not to. The United States lacks anything resembling a functional institutional response to mental illness. Deinstitutionalisation has virtually eliminated inpatient care as a viable healthcare option’ (p 201).

Arguably, political systems that take an essentially pessimistic view of the social bonds of trust,^14^ apportion sacred status to private property,^15^ lionise competition and cultivate grotesque inequalities^16^ would have an interest in maintaining ignorance about what causes mental ill health, unless this is consistent with capital and corporate priorities.^17^ Perhaps the surprise would have been if psychiatrists had not fallen in line.^18^

Historian Jack Pressman, who has studied psychiatric practice in action in *Last Resort: Psychosurgery and the Limits of Medicine* (2002) argues that: ‘Put simply, psychiatry is the management of despair. This is the heart of the psychiatrist's social function, to care for those whose problems have no certain cure or satisfactory explanation’ (quoted by Whooley on p. 27). Another historian, Charles Rosenberg, argues that psychiatry's true role is to preside over the ‘uncanny’ as it is encountered at the boundary of ‘disease, willed behaviour or culpable self-indulgence’ (quoted on p. 220). In Whooley's view, psychiatry is allowed to continue despite its ignorance because, through attention to despair and the uncanny, it ‘allows [the rest of] us the luxury of recoiling from the raving delusions of the schizophrenic, the cheerless gloom of the depressed, the nervous jittering of the anxious, and the tumultuous mood swings of the manic’ (p. 220). If Pressman, Rosenberg and Whooley are right, it should not surprise that psychiatry can sometimes serve as a useful scapegoat. Nor that it may contribute to its scapegoating through denial of its role or premature ‘solutions’.

## Psychiatric expertise

There is one charge against psychiatry by Whooley that cannot simply be deflected onto social factors, namely that it has failed to define the object of its expertise. The anxiety about this uncertainty, a condition akin to but not identical to ignorance, is a key driver for the reinventions. The alarm that this has caused has driven some to proclaim that the object of expertise is the brain. Specifically, White & Zeman^19^ propose that the time has come for psychiatry to merge with neurology. This would be an entirely novel development in the UK and would greatly reduce the scope of the specialty. Others have taken a different view, one that is able to accommodate the affinity between neurology and psychiatry, without indulging the reductionism inherent in the suggestion of identity between the two specialties. An example of this alternative is the ‘enactive model’.^20,21^ This model sees the mind as embodied (brain *and* body), embedded (in its environment), enacted (in purposeful pursuits and relationships) and, according to some proponents, extended in space (through communication and the material media that serve it). Consistent with such views, I have previously proposed that:

‘Unlike neurologists, affect not the brain is the object of psychiatrists’ specialist medical expertise. Defined^a^ as feelings, emotions and agitations, affect integrates human responses and drives brain and body changes, thinking, perceiving, relating and acting. In no particular order, it depends on genes, evolution, culture, physiology, personal experience, social history, chance, meaning, the environment and a sense of self and others’.^22^ [see also^23^]

This proposal allows for the integration of views as diverse as White & Zeman's, Pressman's and Rosenberg's, Whooley's and the proponents of enactive psychiatry. If affect is accepted as the specific object of psychiatry's medical expertise, it adds orders of magnitude of complexity over and above that of the brain, which is probably already too complicated to understand.^24^ To state this is not to ‘blame the object’ for the limited progress that psychiatry, including American psychiatry, has made but to face the facts.

## Discussion

Whooley writes:

‘By no means do these developments suggest that psychiatry is doomed. History is littered with premature declarations and smug prophesies of psychiatry's death, only to see psychiatry resurrected, phoenix like, on the promises of the next new invention’ (p. 196).

As societies change, the very nature of psychiatry will change. Some readjustments will be demanded by changes not only in biomedical evidence but also in society and the state. In this sense, reinvention is not necessarily wrong, though it is if rushed ahead of or against the evidence. Though many will insist that we are less prone to hype on this side of the Atlantic, we are not immune, not least because of the close relationship with our colleagues across the water. If I may be permitted a personal observation in what is intended to be a forum of debate in the journal, I was surprised and alarmed by the messianic tone with which the Royal College of Psychiatrists inaugurated its neuroscience programme to transform psychiatric training in the UK by integrating modern neuroscience, with financial support from the Gatsby Foundation and the Wellcome Trust. The fact that a key guest was a former President of the American Psychiatric Association may have been a contributing factor but the rest is up to us. Some of this tone, not justified by patient outcomes so far, has been carried beyond the College by our members in other public forums.

To meet the challenge psychiatry must forge its distinct identity. This will mean tolerating ignorance and uncertainty, sharing them intelligently with colleagues and the public and engaging creatively rather than concealing or denying them. At the same time continuing to chip away at ignorance and uncertainty where possible. To succeed there is a need to move forward from the rather narrow confines of 20th-century medicine, with its attention to proximate physical mechanisms, and embrace more fully evolutionary approaches,^25^ the social and historical sciences and cultural studies and practices^26^ and scientific pluralism.^27^ This is a tall order, both in the demands it places on the training and continuing professional development curriculum, and the challenges it faces in the often-hostile arenas of professional rivalry and public debate. Nevertheless, it is no more than our patients deserve and keeping an eye on the sociology of ignorance may come in handy too as the gaps in knowledge are unlikely to be sufficiently filled any time soon.

## Conclusions

For psychiatrists in Britain (and beyond), without losing passion for research, including in neuroscience (but also all other relevant areas), nor forsaking clinical and social commitment and hope, there is a need for ongoing recognition of affect as the object of psychiatric expertise, an attitude of humility about the state of advancement of our clinical science, attention to the sociology of ignorance, caution about adopting transatlantic healthcare policies and management techniques, and consistent commimtent to intellectual and clinical pluralism.

## Data Availability

Data availability is not applicable to this article as no new data were created or analysed in this study.

## Footnotes

a. Affect as defined here does not specifically refer to affective disorder nor to affect as it is defined in the psychiatric literature on phenomenology and mental state examination (see Thompson E. Primordial dynamism: emotion and valence. In *Mind in Life: Biology, Phenomenology and the Life Sciences*: 360–81. Harvard University Press, 2010; and Bennett MR, Hacker PMS. Emotion. In *Philosophical Foundations of Neuroscience*: 199–223. Blackwell Publishing, 2007).

## About the author

**George Ikkos** is a consultant liaison psychiatrist in the Department of Psychiatry at the Royal National Orthopaedic Hospital, London, immediate past Chair of the Royal College of Psychiatrists’ History of Psychiatry Special Interest Group (HoPSIG) and former President of the Royal Society of Medicine's Pain Medicine and Psychiatry Sections. He is joint editor of *Mind, State and Society: Social History of Psychiatry and Mental Health in Britain 1960–2010*, which was runner up in the Association of American Publishers’ PROSE 2022 Awards, in the section History of Science, Medicine and Technology (available open access from https://doi.org/10.1017/9781911623793).

## References

[1] Whooley I. On the Heels of Ignorance: Psychiatry and the Politics of Not Knowing. University of Chicago Press, 2018.

[2] Bhugra D, Malik A, Ikkos G. Psychiatry's Contract with Society: Concepts, Controversies and Consequences. Oxford University Press, 2010.

[3] Bourke J. Historical perspectives on mental health and psychiatry. In Mind, State and Society: Social History of Psychiatry and Mental Health in Britain 1960–2010 (eds G Ikkos, N Bouras): 3–12. Cambridge University Press, 2021.

[4] Ikkos G, Dave S. Reflection: 1979 and psychiatry. News and Notes: Newsletter of the Royal College of Psychiatrists’ History of Psychiatry Special Interest Group. 2021; 12: 12–3.

[5] Poole R, Robinson C. Breaking out of the citadel: social theory and psychiatry. BJPsych Bull [Epub ahead of print] 15 Mar 2022. Available from: 10.1192/bjb.2022.17. PMC10214419 35289262

[6] Grünbaum A. The Foundations of Psychoanalysis: a Philosophical Critique. University of California Press, 1984.

[7] Wakefield JC. Klerman's “credo” reconsidered: neo-Kraepelinianism, Spitzer's views, and what we can learn from the past. World Psychiatry 2022; 21: 4–25. 35015356 10.1002/wps.20942 PMC8751581

[8] Shorter E. The international context. In Mind, State and Society: Social History of Psychiatry and Mental Health in Britain 1960–2010 (eds G Ikkos, N Bouras): 13–22. Cambridge University Press, 2021.

[9] Glasby J, Tew J, Fenton S. UK mental health policy and practice. In Mind, State and Society: Social History of Psychiatry and Mental Health in Britain 1960–2010 (eds G Ikkos, N Bouras): 93–102. Cambridge University Press, 2021.

[10] Scull A. UK deinstitutionalisation: neoliberal values and mental health. In Mind, State and Society: Social History of Psychiatry and Mental Health in Britain 1960–2010 (eds G Ikkos, N Bouras): 306–314. Cambridge University Press, 2021.

[11] British Medical Association. Independent Sector Provision in the NHS revisited. BMA, 2019.

[12] Street A. For-profit health care might be damaging population health. Lancet Public Health 2022; 7(7): e576–7. 10.1016/S2468-2667(22)00142-6 35779537

[13] Scambler G. Liberty's command: liberal ideology, the mixed economy and the British welfare state. In Mind, State and Society: Social History of Psychiatry and Mental Health in Britain 1960–2010 (eds G Ikkos, N Bouras): 23–31. Cambridge University Press, 2021.

[14] Hirschman AO. The Passions and the Interests: Political Arguments for Capitalism Before Its Triumph. Princeton University Press, 2013.

[15] Graeber D, Wengrow D. Free people, the origins of culture and the advent of private property (not necessarily in that order). In The Dawn of Everything: A New History of Humanity. Allen Lane, 2021: Ch. 4.

[16] Wilkinson R, Pickett K. The Spirit Level: Why Equality is Better for Everyone. Penguin Books, 2010.

[17] Davies J. Sedated: How Modern Capitalism Created our Mental Health Crisis. Atlantic Books, 2021.

[18] Healy D. The pharmaceutical industry and the standardisation of psychiatric practice. In Mind, State and Society: Social History of Psychiatry and Mental Health in Britain 1960–2010 (eds G Ikkos, N Bouras): 163–70. Cambridge University Press, 2021.

[19] White PD, Zeman AJZ. Time to end the distinction between mental and neurological illnesses. BMJ 2012; 2012: e3454. 10.1136/bmj.e3454 22628005

[20] Fuchs T. Ecology of the Brain. Oxford University Press, 2018.

[21] de Haan S. Enactive Psychiatry. Cambridge University Press, 2020.

[22] Ikkos G. Psychiatric expertise. Br J Psychiatry 2015; 207: 399. 26527665 10.1192/bjp.bp.115.169946

[23] Ikkos G, Bouras N, McQueen D, St. John-Smith P. Medicine, affect and mental health services. World Psychiatry 2010; 9: 35–6. 20148156 10.1002/j.2051-5545.2010.tb00264.x PMC2816910

[24] Marcus G, Freeman J. The Future of the Brain: Essays by the World's Leading Neuroscientists. Princeton University Press, 2015.

[25] Abed R, St John Smith P. Evolutionary Psychiatry: Current Perspectives on Evolution and Mental Health. Cambridge University Press, 2022: in press.

[26] Ikkos G, Bouras N. Epilogue: mind, state, society and ‘our psychiatric future’. In Mind, State and Society: Social History of Psychiatry and Mental Health in Britain 1960–2010 (eds G Ikkos, N Bouras): 393–6. Cambridge University Press, 2021.

[27] Jerotic S, Aftab A. Scientific pluralism is the only way forward for psychiatry. Acta Psychiatr Scand 2021; 143: 537–8. 33988863 10.1111/acps.13298

